# The demise of the randomised controlled trial: bibliometric study of the German-language health care literature, 1948 to 2004

**DOI:** 10.1186/1471-2288-6-30

**Published:** 2006-07-06

**Authors:** Daniel Galandi, Guido Schwarzer, Gerd Antes

**Affiliations:** 1 German Cochrane Center, Department of Medical Biometry and Statistics, University Hospital Freiburg, Stefan-Meier-Str. 26, 79104 Freiburg, Germany

## Abstract

**Background:**

In order to reduce systematic errors (such as language bias) and increase the precision of the summary treatment effect estimate, a comprehensive identification of randomised controlled trials (RCT), irrespective of publication language, is crucial in systematic reviews and meta-analyses. We identified trials in the German general health care literature.

**Methods:**

Eight German language general health care journals were searched for randomised controlled trials and analysed with respect to the number of published RCTs each year and the size of trials.

**Results:**

A total of 1618 trials were identified with a median total number of 43 patients per trial. Between 1970 and 2004 a small but constant rise in sample size from a median number of 30 to 60 patients per trial can be observed. The number of published trials was very low between 1948 and 1970, but increased between 1970 and 1986 to a maximum of 11.2 RCTs per journal and year. In the following time period a striking decline of the number of RCTs was observed. Between 1999 and 2001 only 0.8 RCTs per journal and year were published, in the next three years, the number of published trials increased to 1.7 RCTs per journal and year.

**Conclusion:**

German language general health care journals no longer have a role in the dissemination of trial results. The slight rise in the number of published RCTs in the last three years can be explained by a change of publication language from German to English of three of the analysed journals.

## Background

Approximately more than 10,000 randomised controlled trials (RCTs) – widely acknowledged as the gold standard for the evaluation of medical interventions – are published each year. Systematic reviews of RCTs, such as those disseminated through the Cochrane Collaboration, synthesise the results of individual trials to facilitate the use of external evidence in clinical decision making. The process of systematic reviewing is designed to maximise validity. This is accomplished with a series of steps to minimise bias, such as a comprehensive literature search. Indeed, the exhaustive identification of trials, irrespective of the language of publication, seems important to reduce systematic errors like language bias. Studies investigating the influence of language bias reveal controversial findings. For the comparison of German and English-language trials, a significant trend towards publication of statistically significant results in English-language journals was identified [1]. However, reanalysis of published meta-analysis including both English and non-English language trials found no consistent change in the summary estimates of meta-analyses when excluding non-English language trials. These findings, however, seem to depending on the medical specialty. In complementary medicine several meta-analyses resulted in significantly smaller summary result estimates when excluding non-English language trials [2-4]. Therefore, without consistent empirical evidence supporting the exclusion of non-English language trials, the set of eligible trials for systematic reviews and meta-analysis should be as comprehensive as possible and should therefore not preclude the inclusion of trials because of language reasons.

In order to support reviewers the Cochrane Collaboration organised an international project to manually search the health care literature from many countries for RCTs. The trials identified are added to the Cochrane Controlled Trials Register and thus made available to reviewers.

## Methods

Eight German language general health care journals (and their predecessors) were manually searched for the years 1948 to 2004: *Deutsche Medizinische Wochenschrift*, *Journal of Molecular Medicine *(formerly *Klinische Wochenschrift*), *Medizinische Klinik*, *Medizinische Welt*, *Münchener Medizinische Wochenschrift*, *Swiss Medical Weekly *(formerly *Schweizerische Medizinische Wochenschrift*), *Wiener Medizinische Wochenschrift*, *Zeitschrift für Allgemeinmedizin*.

The sample sizes of the identified trials were extracted and analysed in groups of three consecutive calendar years.

## Results

**Figure 1:**
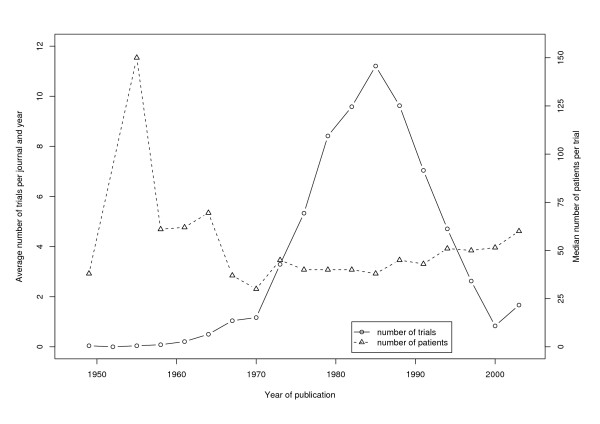
Number and Size of RCTs published in eight German language general health care journals.

1618 RCTs were published as original articles with a median total number of patients of 43 (quartiles 20 and 87). Only 19.3% of the identified trials included more than 100 patients. The number of published trials was very low between 1948 and 1970 and increased between 1970 and 1986 to a maximum of 11.2 RCTs per journal and year (figure 1). In the following time period a striking decline of the number of RCTs was observed. Between 1999 and 2001 only 0.8 RCTs per journal and year were published on average. In the last analysed time period (2002–2004) an increase in the number of published trials can be observed: 1.7 trials per journal and year were published.

Personal communication with editors of the journals resulted in a fairly uniform explanation of this trend: The widespread introduction of using journal impact factors for the allocation of resources has strongly motivated researchers to publish in higher impact journals. As these are almost entirely in English, German general health care journals now rarely receive RCTs for publication.

Concerning the trial size, a slight but steady increase of the number of patients per trial can be observed. The median number of patients per trial rose from 30 in the years 1969 to 1971 to 60 between 2002 and 2004.

## Discussion

The number of RCTs published in the German language general health care literature has declined dramatically between 1986 and 2001. Between 1999 and 2001 eight major German language general health care journals published only 20 RCTs.

This observation confirms developments in other general health care journals, but is in clear contrast to high impact international general and specialist journals where a constant number of RCTs has been observed over past decades [5-7].

The slight increase of the number of published trials in the years 2002 to 2004 can (partially) be explained by changes of publication language from German to English in three of the analysed journals (more than 50% of the 39 RCTs published between 2002 and 2004 were in English language).

The sample size of trials identified in German language general health care journals was small with an increase in the more recent volumes. Although the trial size increased, it remains even in recent years rather small with a median number of 60 patients per trial. The average sample size of RCTs published in German appears to be comparable to other general health care journals and specialist journals, however, trials published in international journals of high impact are considerably larger [5,7]. The proportion of RCTs with 100 or more patients published in the *BMJ *and the *Annals of Internal Medicine *in the years 1995–1997 was 76% compared to 19.3% in the investigated German language journals [5].

The observed decline of RCTs published in German language general health care journals raises the question whether the number of trials is declining or whether trials nowadays are more frequently not published or published elsewhere. Currently German ethics committees do not know the exact number of approved RCTs because their records do not distinguish between different study designs. We can only speculate whether an increasing proportion of these trials is never published, or where these trials have been published in recent years. What can be concluded is that the leading general health care journals in the German language area have abandoned their role in the dissemination of RCTs. However, the change of the publication language from German to English (as in the Schweizerische Medizinische Wochenschrift – now swiss Medical Weekly) resulted in a contrasting development with an increase of the number of published RCTs.

## Conclusion

Allocation of funding resources and the scientific career depend to a great extent on the value of the scientific output (measured by impact factor points) of an institution or an individual researcher. The result is an increasingly fierce competition for publication in high impact journals, with high rejection rates, influencing the chance to get manuscripts published in due time. German-language RCTs, and those from comparable non-English language countries, face far more obstacles to publication than a decade ago when they were easily submitted to German-language or other non-English language journals, with a good chance of being accepted for publication. It is unclear how these changes in dissemination patterns affect the validity of systematic reviews and meta-analyses, and, generally, the transfer of knowledge from research into practice. Prospective trial registration, frequently stipulated but not implemented universally, would allow tracking trials from ethics committee approval to publication and help prevent under- and overreporting [8]. This study adds another piece of evidence to the urgent need for more transparency in the dissemination process, as it is intended by the present initiative of WHO [9]. In this initiative WHO has taken the lead to establish a standardized, global framework for the prospective registration of clinical trials as e.g. required by the International Committee of Medical Journal Editors (ICMJE) to reduce biases in the reporting and publication process of clinical trials and thus improve the base for decision making in health care [10].

## Abbreviations

RCT: randomized controlled trial

## Competing interests

The author(s) declare that they have no competing interests.

## Authors' contributions

DG: guarantor, handsearching, data extraction, writing the paper

GS: statistical analysis, diagram

GA: project coordination, advice concerning the design of the research project the presentation of the data and writing the paper

## Pre-publication history

The pre-publication history for this paper can be accessed here:


